
JOURNAL INFORMATION
==============================
NLM Title Abbreviation: ScientificWorldJournal
Journal ID: ScientificWorldJournal
NLM Journal ID: 101131163
Journal ID: TSWJ

The Scientific World Journal

ISSN: 2356-6140
EISSN: 1537-744X
Publisher: Wiley

ARTICLE INFORMATION
==============================
PMCID: PMC6084123
PMID: 30147530
DOI: 10.1100/tsw.2001.181
Article ID: 384191
Article version: 1
Subjects: Short Report

DNA Replication Arrest and P53-Dependent and P53-Independent Cell Death Pathways in Xeroderma Pigmentosum Variant Cells

Cleaver James E. *
Crowley Eileen
Kashani-Sabet Mohammed
Limoli Charles L.
UCSF Cancer Center and Depts of Dermatology and Radiation Oncology, University of California, San Francisco, CA 94143, USA
*James E. Cleaver: jcleaver@cc.ucsf.edu
Publication date: 2001
Electronic publication date: 2001 Dec 6
Volume: 1
First page: 60
Last page: 60
Copyright: Copyright © 2001 James E. Cleaver et al.
Copyright year: 2001
License: This is an open access article distributed under the Creative Commons Attribution License, which permits unrestricted use, distribution, and reproduction in any medium, provided the original work is properly cited.
License URL: https://creativecommons.org/licenses/by/3.0/

Funding: National Institute of Environmental Health Sciences 1 RO1 ES 08061 Ellison Medical Foundation

==============================
Miami *Nature Biotechnology* Short Reports
*TheScientificWorld* (2001) 1 (S3), 60SR
ISSN 1532-2246; DOI 10.1100/tsw.2001.181

### DNA REPLICATION ARREST AND P53-DEPENDENT AND P53-INDEPENDENT CELL DEATH PATHWAYS IN XERODERMA PIGMENTOSUM VARIANT CELLS

James E. Cleaver*, Eileen Crowley, Mohammed Kashani-Sabet, and Charles L. Limoli

UCSF Cancer Center and Depts of Dermatology and Radiation Oncology, University of California, San Francisco,CA 94143
* jcleaver@cc.ucsf.edu.

**INTRODUCTION.** Xeroderma pigmentosum variant (XPV) cells lack the low-fidelity DNA polymerase, pol $\eta$, and as a result cannot replicate UV damaged DNA and experience prolonged arrest in the S phase (1, 2). Primary XPV fibroblasts and XPV fibroblasts transformed by HPV16(E6/E7) do not show early UV-induced apoptosis, but remain arrested for up to 48 hr without evidence of cell death (3). XPV cells transformed by SV40, however, show very early apoptosis, which is further enhanced by caffeine, and a large fraction of the cell population detaches from the substrate within 10-20 hr (3, 4). In the presence of caffeine, apoptosis is detectable in 1-2 hr. Transfection of SV40 transformed cells with the XPV gene, *hRad30A*, on a CMV promoter suppressed UV-induced apoptosis. Normal cells show parallel apoptotic behavior, but at a lower level. Paradoxically, when cells are assayed for colony formation after UV irradiation, the XPV cells transformed by HPV16,E6/E7) are much more sensitive than SV40 transformed or primary cells (3). Therefore the degree of apoptosis is no measure of long-term survival, and a non-apoptotic pathway of cell death, caused by DNA replication arrest, is associated with higher lethality than the apoptotic pathway.

**METHOD.** It seemed that the major difference between these three cellular states was the status of p53: primary cells show low levels that are transiently stabilized by UV irradiation; SV40 transformed cells contain high levels of p53 stabilized in complexes with large T; HPV16(E6/E7) transformed cells contain no p53 due to ubiquitination by the E6 gene product. We decided that a direct test of this hypothesis could be achieved if we could specifically eliminate p53 from SV40 transformed cells. Apoptosis was quantified by determining the fraction of cells detached from their substrates 16-20 hr after 6.5 $J.m^{-2}$ UV light. Only detached cells showed caspase-3 activation and PARP cleavage (4).

**RESULTS.** Inhibition of protein synthesis with cycloheximide or inhibition of p53 transactivating activity with pifithrin-$\alpha$ had no effect on UV-induced apoptosis. Inhibition of apoptosis by the caspase-3 peptide inhibitor zVAD resulted in irradiated cells remaining attached to the substrate. These results suggested that UV-induced apoptosis was due to a direct effect of the p53 protein in SV40 transformed cells. We designed a test of this hypothesis by synthesizing several ribozymes (sense and antisense) designed to cleave p53 mRNA. These ribozymes were expressed from the high copy number episomal vector pREP4 that uses a RSV promoter. They were designed to target positions 13080, 18610,18757, and 18910 in the p53 gene. We have previously successfully expressed a pol $\zeta$ ribozyme in this vector (5). Western blots were carried out to determine whether p53 protein was eliminated from SV40 transformed cells using these ribozyme sequences. The first ribozyme sequence (3'-TT CTA

CA AAG CAG GAG TGC CTG AGT AGT CAAC GGT-5’) was ineffective and cultures all retained similar high levels of p53 protein. Other ribozyme sequences are under investigation at the present time.

**DISCUSSION.** Successful development of a p53 ribozyme by high level episomal expression should allow a very specific test of p53 functions in SV40 transformed and other cell types. We have shown, for example, that recovery of DNA replication can occur either by a replicative bypass mechanism (pol η, κ, ζ and others) or a recombination mechanism (hMre11/hRad50/Nbs1) involving the activity of p53 (4, 5). Specific experiments to understand how these choices are determined by various functions of p53 will be possible using the ribozyme approach.

**ACKNOWLEDGEMENT.** This work was supported by NIEHS grant 1 RO1 ES 08061 and a grant from the Ellison Foundation for Research on Aging, and generous support from the XP Society.

**REFERENCES.**

1. Johnson, R.E., Kondratick, C.M., Prakash, S., and Prakash, L. (1999) Science 264, 263-266
2. Masutani, C., Kusumoto, R., Yamada, A., Dohmae, N., Yokol, M., Yuasa, M., Araki, M., Iwa, S., Takio, K., and Hanoaka, F. (1999) Nature 399, 700-704
3. Cleaver, J.E., Afzal, V., Feeney, L., McDowell, M., Sadinski, W., Volpe, J.P.G., Busch, D., Yu, Y., Nagasawa, H., and Little, J.B. (1999) Cancer Res. 59, 1102-1108
4. Limoli, C.L., Giedzinski, E., Morgan, W.F., and Cleaver, J.E. (2000) Proc. Natl. Acad. Sci. U S A 97, 939-7946
5. Cleaver, J.E., Karplus, K., Kashani-Sabet, M., and Limoli, C.L. (2000) Mutat. Res. (in press)
